# Glioblastoma and radiotherapy: A multicenter AI study for Survival Predictions from MRI (GRASP study)

**DOI:** 10.1093/neuonc/noae017

**Published:** 2024-01-29

**Authors:** Alysha Chelliah, David A Wood, Liane S Canas, Haris Shuaib, Stuart Currie, Kavi Fatania, Russell Frood, Chris Rowland-Hill, Stefanie Thust, Stephen J Wastling, Sean Tenant, Catherine McBain, Karen Foweraker, Matthew Williams, Qiquan Wang, Andrei Roman, Carmen Dragos, Mark MacDonald, Yue Hui Lau, Christian A Linares, Ahmed Bassiouny, Aysha Luis, Thomas Young, Juliet Brock, Edward Chandy, Erica Beaumont, Tai-Chung Lam, Liam Welsh, Joanne Lewis, Ryan Mathew, Eric Kerfoot, Richard Brown, Daniel Beasley, Jennifer Glendenning, Lucy Brazil, Angela Swampillai, Keyoumars Ashkan, Sébastien Ourselin, Marc Modat, Thomas C Booth

**Affiliations:** School of Biomedical Engineering & Imaging Sciences, King’s College London, London, UK; School of Biomedical Engineering & Imaging Sciences, King’s College London, London, UK; School of Biomedical Engineering & Imaging Sciences, King’s College London, London, UK; Guy’s and St. Thomas’ NHS Foundation Trust, London, UK; Institute of Psychiatry, Psychology & Neuroscience, King’s College London, London, UK; Leeds Teaching Hospitals NHS Trust, Leeds, UK; Leeds Teaching Hospitals NHS Trust, Leeds, UK; Leeds Teaching Hospitals NHS Trust, Leeds, UK; Hull University Teaching Hospitals NHS Trust, England, UK; University College London Hospitals NHS Foundation Trust, London, UK; Institute of Neurology, University College London, London, UK; Nottingham University Hospitals NHS Trust, Nottingham, UK; Precision Imaging Beacon, School of Medicine, University of Nottingham, Nottingham, UK; University College London Hospitals NHS Foundation Trust, London, UK; Institute of Neurology, University College London, London, UK; The Christie NHS Foundation Trust, Withington, Manchester, UK; The Christie NHS Foundation Trust, Withington, Manchester, UK; Nottingham University Hospitals NHS Trust, Nottingham, UK; Radiotherapy Department, Imperial College Healthcare NHS Trust, London, UK; Institute for Global Health Improvement, Imperial College London, London, UK; Radiotherapy Department, Imperial College Healthcare NHS Trust, London, UK; Institute for Global Health Improvement, Imperial College London, London, UK; Guy’s and St. Thomas’ NHS Foundation Trust, London, UK; Oncology Institute Prof. Dr. Ion Chiricuta, Cluj-Napoca, Romania; Buckinghamshire Healthcare NHS Trust, Amersham, UK; Guy’s and St. Thomas’ NHS Foundation Trust, London, UK; King’s College Hospital NHS Foundation Trust, London, UK; Guy’s and St. Thomas’ NHS Foundation Trust, London, UK; School of Biomedical Engineering & Imaging Sciences, King’s College London, London, UK; Department of Radiology, Mansoura University, Mansoura, Egypt; School of Biomedical Engineering & Imaging Sciences, King’s College London, London, UK; King’s College Hospital NHS Foundation Trust, London, UK; Guy’s and St. Thomas’ NHS Foundation Trust, London, UK; Brighton and Sussex University Hospitals NHS Trust, England, UK; Brighton and Sussex University Hospitals NHS Trust, England, UK; Lancashire Teaching Hospitals NHS Foundation Trust, England, UK; Lancashire Teaching Hospitals NHS Foundation Trust, England, UK; The Royal Marsden NHS Foundation Trust, London, UK; Newcastle upon Tyne Hospitals NHS Foundation Trust, England, UK; Leeds Teaching Hospitals NHS Trust, Leeds, UK; School of Medicine, University of Leeds, Leeds, UK; School of Biomedical Engineering & Imaging Sciences, King’s College London, London, UK; School of Biomedical Engineering & Imaging Sciences, King’s College London, London, UK; School of Biomedical Engineering & Imaging Sciences, King’s College London, London, UK; Guy’s and St. Thomas’ NHS Foundation Trust, London, UK; Maidstone and Tunbridge Wells NHS Trust, Kent, UK; Guy’s and St. Thomas’ NHS Foundation Trust, London, UK; Guy’s and St. Thomas’ NHS Foundation Trust, London, UK; Institute of Psychiatry, Psychology & Neuroscience, King's College London, London, UK; King’s College Hospital NHS Foundation Trust, London, UK; School of Biomedical Engineering & Imaging Sciences, King’s College London, London, UK; School of Biomedical Engineering & Imaging Sciences, King’s College London, London, UK; School of Biomedical Engineering & Imaging Sciences, King’s College London, London, UK; King’s College Hospital NHS Foundation Trust, London, UK

**Keywords:** artificial intelligence, deep learning, glioblastoma, magnetic resonance imaging, survival

## Abstract

**Background:**

The aim was to predict survival of glioblastoma at 8 months after radiotherapy (a period allowing for completing a typical course of adjuvant temozolomide), by applying deep learning to the first brain MRI after radiotherapy completion.

**Methods:**

Retrospective and prospective data were collected from 206 consecutive glioblastoma, isocitrate dehydrogenase -wildtype patients diagnosed between March 2014 and February 2022 across 11 UK centers. Models were trained on 158 retrospective patients from 3 centers. Holdout test sets were retrospective (*n* = 19; internal validation), and prospective (*n* = 29; external validation from 8 distinct centers). Neural network branches for *T*_2_-weighted and contrast-enhanced *T*_1_-weighted inputs were concatenated to predict survival. A nonimaging branch (demographics/MGMT/treatment data) was also combined with the imaging model. We investigated the influence of individual MR sequences; nonimaging features; and weighted dense blocks pretrained for abnormality detection.

**Results:**

The imaging model outperformed the nonimaging model in all test sets (area under the receiver-operating characteristic curve, AUC *P* = .038) and performed similarly to a combined imaging/nonimaging model (*P > *.05). Imaging, nonimaging, and combined models applied to amalgamated test sets gave AUCs of 0.93, 0.79, and 0.91. Initializing the imaging model with pretrained weights from 10 000s of brain MRIs improved performance considerably (amalgamated test sets without pretraining 0.64; *P* = .003).

**Conclusions:**

A deep learning model using MRI images after radiotherapy reliably and accurately determined survival of glioblastoma. The model serves as a prognostic biomarker identifying patients who will not survive beyond a typical course of adjuvant temozolomide, thereby stratifying patients into those who might require early second-line or clinical trial treatment.

**Key Points**

- A deep learning model predicted post-radiotherapy survival of glioblastoma from MRIs.
- An imaging model was generalizable on internal and prospective external test data.
- Performance was considerably better when initial weights were pretrained on 10 000s of MRIs.

**Importance of the Study**

A deep learning model that used MRI images after radiotherapy, and that was pretrained on 10 000s of brain MRIs, reliably and accurately determined survival of isocitrate dehydrogenase (IDH) wildtype glioblastoma patients after radiotherapy.

Glioblastoma is the most aggressive adult primary brain cancer.^1^ MRI plays a key role in diagnosis, treatment planning, and treatment response assessment.^2^ MRI images can also act as prognostic biomarkers with studies predicting survival from preoperative MRIs using classical^3^ and deep^4,5^ machine learning models. However, by the time radiotherapy finishes, considerable intervention potentially confounds survival predictions obtained at the preoperative time point. Survival predictions from images obtained after radiotherapy could be more accurate. To our knowledge, machine learning has not been applied to the first MRI images after radiotherapy completion to identify patients who will not survive beyond a typical course of adjuvant temozolomide (TMZ). In this scenario, an accurate and generalizable prognostic biomarker would stratify patients into those requiring early second-line treatment or clinical trial enrollment. Additionally, all subsequent tumor boards held during the course of adjuvant TMZ would have an accurate *a priori* survival prediction, therefore improving management decision confidence. This is relevant as often follow-up imaging findings are nonspecific and treatment response assessment is not definitive; even when findings are specific, utility is based on low-level evidence.^6^

Optimal treatment involves surgical resection, followed by radiotherapy with concomitant TMZ, then adjuvant TMZ^7,8^ (see Supplementary Appendix A for an illustration of treatment and imaging pathways). Modified treatment may be planned for patients who are elderly or have tumors in eloquent areas, or who cannot tolerate optimal treatment.^1,2^ This often includes a shorter course and lower dose of radiotherapy, where a longer course of adjuvant chemotherapy may be prescribed. While 99% of US patients ≥ 66 years undergoing post-surgical treatment receive radiotherapy, just 57% receive TMZ.^9^ Only 34% of UK patients between 20 and 70 years complete optimal treatment.^1^ To inform patient management, MRIs are often performed after initial surgery, during radiotherapy planning, and at 2–3 monthly intervals (or if clinically deteriorating) during subsequent follow-up.^2,9–11^ However imaging studies, including those predicting survival,^3–5,12^ typically sample patients only from the optimally-treated population limiting biomarker applicability. The unmet need to improve outcomes of patients undergoing modified treatment, highlighted at national strategic level^13,14^ (and study stakeholder feedback; Supplementary Appendix B), motivated our biomarker design to be applicable to both optimal and modified treatment populations.

This study aimed to apply deep learning to the first brain MRI after radiotherapy, in glioblastoma, IDH-wildtype^15^ patients undergoing optimal or modified treatment, to predict survival at 8 months after completing radiotherapy (a period allowing for completion of a typical course of adjuvant TMZ). For imaging-based biomarkers to be valuable in the clinic, it is rational that predictions should either be more accurate than those derived from freely available nonimaging information known to be associated with poorer patient survival, or are enhanced when combined. We hypothesized that prediction based on imaging would outperform prediction using only available nonimaging information (demographic, pathological, and treatment-related variables).

## Methods

Study reporting followed the Checklist for Artificial Intelligence in Medical Imaging (CLAIM).^16^ The UK’s Health Research Authority provided ethical approval (ref:18/LO/1873); data were anonymized before analyses.

### Patient Characteristics

***Patient cohort***.—This study included consecutive retrospective and prospective data from 11 ZGBM (zeugmatography for glioblastoma) consortium centers,^17^ with diagnoses between March 2014 and February 2022 (a CONSORT diagram displaying the flow of patients included in analyses is presented in Supplementary Appendix C). The study was pragmatic; imaging regimens were not standardized and were expected to vary over centers and time.^18^ Inclusion criteria consisted of adults diagnosed with glioblastoma, IDH-wildtype^15^; who underwent radiotherapy after first surgery; and subsequent MRI with contrast-enhanced *T*_1_-weighted (T1c) and *T*_2_-weighted (T2) sequences; and could be identified as being deceased or not at 8 months post-radiotherapy (labeled as short-term or long-term survival, respectively).

Long-term survivors who received second-line or trial treatment within 8 months were excluded to prevent confounding from that treatment. As the classifier is designed to help decision-making on expediting early trial or second-line treatment, we excluded those rare patients whose first post-radiotherapy MRI occurred either after second-line treatment started (to prevent confounding), or beyond 24 weeks (arbitrary time threshold). T1c and T2 sequences were selected to maximize the clinical applicability of developed models, as these are acquired in routine clinical settings^18^ and were available for all patients in this cohort. It should be noted that other MR sequences such as FLAIR are informative images and are commonly acquired. However, 18.5% (23/124) of patients in the largest retrospective cohort (the KCH cohort) reported here did not have FLAIR imaging during the first post-radiotherapy MRI study.

Of 206 patients included (Table 1), 64 (31.1%) were short-term survivors (<8 months survival). The amalgamated test set consisted of all prospective external data (henceforth *prospective test set*; *n* = 29) and 10.7% of holdout retrospective data (*retrospective test set*; *n* = 19/177). Stratified sampling into training and test sets was performed on retrospective data to avoid bias from imbalances in survival outcome and MRI acquisition dimensionality across sites. We sampled 89.3% of retrospective patients (*n* = 158/177) as the training set, and the remaining were held out for testing. No further variables were stratified due to low patient numbers after controlling for 3 variables. Description of sample sizes and sampling error associated with survival outcome, acquisition dimension, and variables associated with survival (including age, initial surgery type, and MGMT methylation status) are presented in Appendix D1.

**Table 1.** Patient Cohort Described by Center, Data Collection Period (Retrospective/Prospective), Outcome (Short/Long-Term Survival), and MRI Acquisition Dimension (2D/3D). The Amalgamated Holdout Test Set Consists of a *Prospective Test Set* (All Patients from 8 Prospective Centers, *n* = 29) and a *Retrospective Test Set* (*n* = 19 Patients from 2 Centers; of Which KCH *n* = 13, LTHT *n* = 6)

		Survival Outcome		T1c Acquisition Dimension	
Centre	*N* Total (% of Dataset)	Short-Term *N* (% of Center)	Long-Term *N* (% of Center)	2D *N* (% of Center)	3D *N* (% of Center)
**Retrospective Data Collection**					
** **KCH	124 (70.1%)	35 (28.2%)	89 (71.8%)	39 (31.5%)	85 (68.5%)
** **LTHT	47 (26.6%)	14 (29.8%)	33 (70.2%)	41 (87.2%)	6 (12.8%)
** **UCLH	6 (3.4%)	2 (33.3%)	4 (66.7%)	1 (16.7%)	5 (83.3%)
*Total*	*177 (85.9%)*	*51 (28.8%)*	*126 (71.2%)*	*81 (45.8%)*	*96 (54.2%)*
**Prospective Data Collection**					
** **BSUH	2 (1.0%)	1 (50.0%)	1 (50.0%)	2 (100.0%)	0 (0.0%)
** **Christie	7 (3.4%)	1 (14.3%)	6 (85.7%)	7 (100.0%)	0 (0.0%)
** **HEY	8 (3.9%)	6 (75.0%)	2 (25.0%)	5 (62.5%)	3 (37.5%)
** **ICHT	4 (1.9%)	1 (25.0%)	3 (75.0%)	1 (25.0%)	3 (75.0%)
** **LTHTR	2 (1.0%)	1 (50.0%)	1 (50.0%)	2 (100.0%)	0 (0.0%)
** **Marsden	1 (0.5%)	0 (0.0%)	1 (100.0%)	0 (0.0%)	1 (100.0%)
** **NUH	1 (0.5%)	0 (0.0%)	1 (100.0%)	1 (100.0%)	0 (0.0%)
** **NUTH	4 (1.9%)	3 (75.0%)	1 (25.0%)	4 (100.0%)	0 (0.0%)
*Total*	*29 (14.1%)*	*13 (44.8%)*	*16 (55.2%)*	*22 (75.9%)*	*7 (24.1%)*
***Total***	*206 (100%)*	*64 (31.1%)*	*142 (68.9%)*	*103 (50%)*	*103 (50%)*

*KCH*: King’s College Hospital NHS Foundation Trust; patients were treated across KCH, Guy’s and St Thomas’ NHS Foundation Trust, and the Kent Oncology Centre. *LTHT*: Leeds Teaching Hospitals NHS Trust. *UCLH*: University College London Hospitals NHS Foundation Trust. *BSUH*: Brighton and Sussex University Hospitals NHS Trust. *Christie*: The Christie NHS Foundation Trust. *HEY*: Hull University Teaching Hospitals NHS Trust. *ICHT*: Imperial College Healthcare NHS Trust. *LTHTR*: Lancashire Teaching Hospitals NHS Foundation Trust. *Marsden*: The Royal Marsden NHS Foundation Trust. *NUH*: Nottingham University Hospitals NHS Trust. *NUTH*: Newcastle upon Tyne Hospitals NHS Foundation Trust.

***Covariates***.—Nonimaging information associated with poorer survival includes patients who are older (>60 years), or have tumors which are unmethylated, have minimal O^6^-methylguanine-DNA methyltransferase (MGMT) methylation, are deep-seated (midbrain/thalamus/callosum) or have undergone biopsy alone.^19–22^ These, and other demographic, histologic, tumor-related, and prior treatment variables were included in nonimaging models (Table 2). Of available data, the Eastern Cooperative Oncology Group (ECOG)^23^ performance status did not differ between short-term and long-term survivors within KCH training patients (*P > *.05) (median = 0; range = 0–2); such formal assessments are not regularly administered and, when applied, can be subjective in choice and nature.^6^ Performance status was therefore excluded. Mean/mode imputation was used for missing data; labels were added identifying imputed inputs. Numeric attributes were standardized to unit variance using training data. Categoric variables were one-hot encoded. MGMT methylation was handled in 2 ways. First, a numeric variable identified the MGMT methylation percentage. Second, 3 distinct categoric variables were added identifying if patients had methylated, unmethylated, or unknown (missing) MGMT methylation status. Distributions of nonimaging variables were compared between short-term and long-term survivors using Mann–Whitney *U* and Chi-squared tests. Significance was set at *P* ≤ .05 for all analyses.

**Table 2.** Patient Characteristics Described Overall (All Patients), and by Survival Outcome (Short-Term or Long-Term Survivors Defined as ≤ or >8 Months Survival from the End of Radiotherapy, Respectively)

Variable	All Patients	Short-Term Survivors	Long-Term Survivors	*P Value* ^a^
	(*n* = 206)	(*n* = 64)	(*n* = 142)	
**Survival**				
Deceased date, *n* (%)				–
** **Known	183 (88.8%)	64 (100.0%)	119 (57.8%)	
** **Unknown	23 (11.2%)	0 (0.0%)	23 (11.2%)^b^	
Survival time from end of radiotherapy, in weeks				–
** **Mean (SE^c^)	73.1 (4.1)	21.9 (1.1)	96.2 (4.8)	
**Demographic Variables**				
Sex, *n* (%)				.12
** **Female	72 (35.0%)	17 (26.6)	55 (38.7%)	
** **Male	134 (65.0%)	47 (73.4%)	87 (61.3%)	
Age at first diagnosis, in y				.28
** **Mean (SE)	57.4 (0.7)	59.0 (1.13)	56.7 (0.9)	
** **Unknown, *n* (%)	1 (0.5%)	0 (0.0%)	1 (0.7%)	
**Histologic Variables**				
MGMT^d^ status, *n* (%)				.13
** **Methylated	87 (42.2%)	21 (32.8%)	66 (46.5%)	
** **Unmethylated	114 (55.3%)	42 (65.6%)	72 (50.7%)	
** **Unknown	5 (2.4%)	1 (1.6%)	4 (2.8%)	
MGMT methylation percentage				.04
** **Mean (SE)	16.4 (1.4)	10.9 (1.9)	18.7 (1.8)	
** **Unknown, *n* (%)	26 (12.6%)	10 (26.6%)	16 (12.0%)	
**Tumor location**				
Deep-seated location^e^, *n* (%)				.21
** **Deep-seated	25 (12.1%)	11 (17.2%)	14 (9.9%)	
** **Not deep-seated	181 (87.9%)	53 (82.8%)	128 (90.1%)	
**Treatment Variables**				
Surgery type, *n* (%)				<.001
** **Biopsy-only	48 (23.3%)	25 (39.1%)	23 (16.2%)	
** **Resection	158 (76.7%)	39 (60.9%)	119 (83.8%)	
Radiotherapy dose, *n* (%)				.03
** **Stupp dose^f^	160 (77.7%)	43 (67.2%)	117 (82.4%)	
** **Reduced dose	36 (17.5%)	18 (28.1%)	18 (12.7%)	
** **Not documented	10 (4.9%)	3 (4.7%)	7 (4.9%)	
Concomitant temozolomide dose, *n* (%)				.001
** **Stupp dose	126 (61.2%)	29 (45.3%)	97 (68.3%)	
** **Reduced dose	26 (12.6%)	15 (23.4%)	11 (7.7%)	
** **No temozolomide	23 (11.2%)	11 (17.2%)	12 (8.5%)	
** **Not documented	31 (15.0%)	9 (14.1%)	22 (15.5%)	
**Imaging-Related Variables**				
Duration between radiotherapy and input MRI, in weeks^g^				.02
** **Mean (SE)	8.7 (0.3)	7.5 (0.6)	9.2 (0.4)	
Scanner manufacturer, *n* (%)				.43
** **General electric	55 (26.7%)	14 (21.9%)	41 (28.9%)	
** **Mirada	1 (0.5%)	0 (0.0%)	1 (0.7%)	
** **Philips	8 (3.9%)	3 (4.7%)	5 (3.5%)	
** **Siemens	141 (68.4%)	46 (71.9%)	95 (66.9%)	
** **Toshiba	1 (0.5%)	1 (1.6%)	0 (0.0%)	
T1c dimension, *n* (%)				.45
** **2D	103 (50.0%)	35 (54.7%)	68 (47.9%)	
** **3D	103 (50.0%)	29 (45.3%)	74 (52.1%)	

^a^ *P* values reflect the statistical significance of distributions for demographic, histologic, tumor location, treatment-related, and imaging-related variables between short-term and long-term survivors, calculated with Mann–Whitney *U* and Chi-squared tests.

^b^Albeit known to be alive beyond 8 months post-radiotherapy.

^c^ *SE*: standard error.

^d^ *MGMT*: O^6^-methylguanine-DNA methyltransferase methylation. Methylated status refers to an MGMT methylation percentage above a 10% cutoff point.

^e^Deep-seated location: tumor infiltrates the midbrain, thalamus, or callosum.

^f^Stupp dose: radiotherapy dose of 60 Gy delivered in 30 fractions.

^g^A histogram showing the time between radiotherapy and the first MRI images after radiotherapy completion is presented in Appendix B2.

### Nonimaging Models

Machine learning models (logistic regression, linear and Gaussian support vector classifiers (SVC), and decision tree classifiers) were applied to training data with sequential feature selection using scikit-learn.^24^ Tuned parameters were logistic regression and SVC regularization parameters, gaussian SVC gamma coefficients, and decision tree gini and entropy criteria. We also applied fully connected neural networks to nonimaging features alone (Supplementary Appendix E1).

### Imaging and Combined Models

Whole-brain T1c and T2 images were coregistered and minimally preprocessed using a similar approach to that for a model^25,26^ applied for pretraining. MRI inputs were converted from DICOM into NIfTI format. T2 scans were registered to the corresponding T1c image for each patient and MRI study. Images were resampled to common voxel sizes (1 mm^3^), and subsequently cropped or padded to a final 3D array of shape 130 × 130 × 130 for inputs to deep learning models. Resampling was performed to address differences in slice thickness and spacing between images. Cropping/padding was performed to preserve the aspect ratios of images when resizing to the final shape. Image preprocessing was conducted with niftyreg^27^ and MONAI.^28^

**Figure 1.**
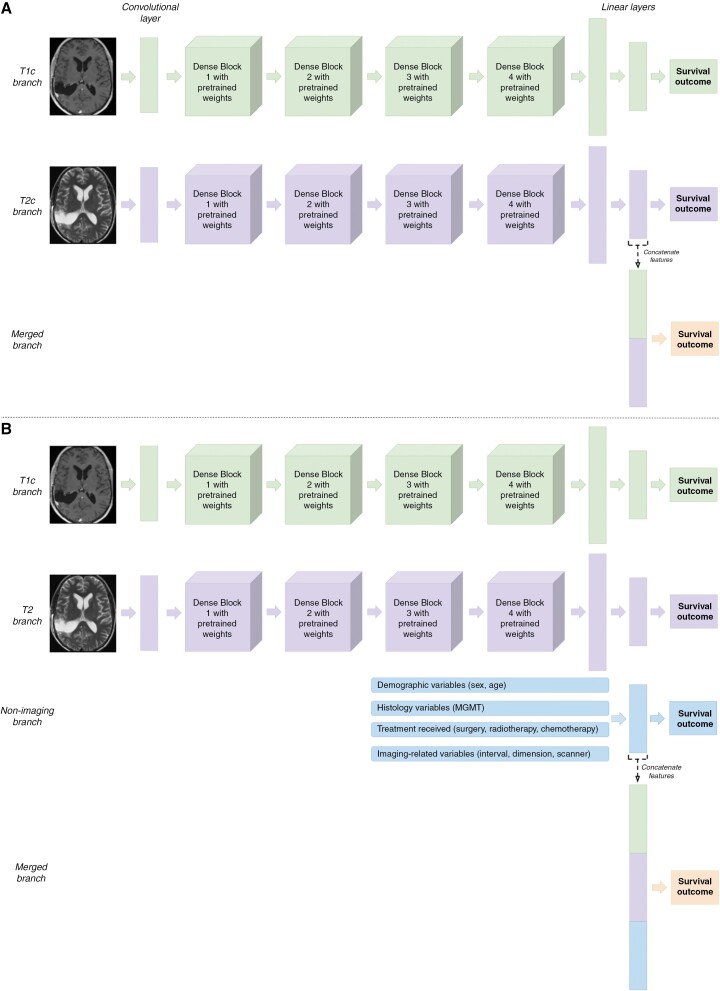
Architectures for dense neural networks. (a) Imaging model: The model inputs whole brain contrast-enhanced *T*_1_-weighted sequences, and *T*_2_-weighted sequences as separate branches (T1c and T2 branches). These are passed through dense blocks with pretrained weights. Outputs are flattened and reduced before feature concatenation. Predictions are obtained from the merged linear layer (concatenating vectors from T1c and T2 branches). (b) *Combined model*: Modified version of the architecture with an additional branch consisting of nonimaging inputs and linear layers. For illustrative purposes, 3D MR volumes are shown as 2D images and 4D dense blocks as 3D representations.

***Network Architectures***.—Model architectures (Figure 1a) were modified from DenseNet121^29^ and abnormality detection models^25,26^ (Supplementary Appendix E2 describes an alternative architecture considered). Input images were the final 3D array of shape 130 × 130 × 130. Dense blocks were initialized with weights pretrained on a large dataset containing all neurological abnormalities (10 695 and 50 523 T1c and T2 scans, respectively). The T1c-branch has 4 pretrained dense blocks. Outputs are flattened to a 1 × 1920-dimensional vector via pooling, then passed through 2 linear layers (providing prediction probabilities). The T2-branch performs the analogous process for T2 inputs. Outputs from the first linear layer per branch are concatenated (*merged branch*); this vector is passed through a linear layer that outputs a 1 × 2-dimensional vector with prediction probabilities. Since each branch can predict survival separately, distinct loss functions are applied per branch. Outputs from the merged branch were selected as final predictions.

A separate *combined* model adds a nonimaging branch with 1 × 27-dimensional inputs alongside the T1c and T2 branches (Fig.1b); the nonimaging branch of this combined model additionally included the duration between radiotherapy completion and imaging (Table 2). The merged prediction is obtained by concatenating T1c, T2, and nonimaging vectors.

Final (hyper-)parameters of model training and tuning (Supplementary Appendix E3) were selected by mean validation area under the receiver-operating characteristic curve (AUC) across training folds. All models incorporating imaging were developed with both PyTorch^30^ and the PyTorch-based MONAI^28^ framework.

### Test Set Analysis

Five-fold cross-validation was used on training data (stratified by outcome/dimension/center) (Supplementary Appendix D1). To determine generalizability, individual imaging, nonimaging, and combined models were trained on all training data and assessed on holdout test data.

To check for dependencies between features and outcomes, a permutation test was performed with test set inputs per patient shuffled before determining model performance. Ablation studies were conducted to investigate the relative importance of individual branches and the use of pretrained weights. Model explainability was further pursued using a guided backpropagation approach^25^ modified to obtain saliency maps from merged branch weights and multiple sequences. As an overview, guided backpropagation is intended to highlight regions of input images which, if modified slightly, would change predictions obtained from the model. The method returns gradient arrays that match the dimensions of the original 3D input images. For visualization purposes of volumetric saliency maps, axial slices that most contributed to model survival predictions were automatically selected and presented, following the methodology reported by Wood et al. (2022).^25^

The primary outcome measure was AUC. We used DeLong’s test to compare model performances (pROC R package).^31^ Subgroup analyses considered retrospective/prospective collection, surgery type, age (>60 years), sex, and acquisition dimension. The code is available at https://github.com/lyshc/glioblastoma-survival-classifier.

## Results

### Patient Characteristics

The dataset included 206 consecutive patients (Tables 1 and 2; Supplementary Appendix C). The mean age was 57.4 (SD: 10.6); 72 patients were female and 134 were male. Missing data for at least 1 variable (age, MGMT status, MGMT methylation percentage, radiotherapy dose, or TMZ dose) were noted in 57/206 (27.7%) patients. For 13 patients, the MGMT status was known while the exact methylation percentage was missing (methylated, *n* = 7; unmethylated, *n* = 6); the percentage was imputed based on the mean percentage for other patients with the same methylation status.

Longer survival was associated with tumors that have higher MGMT methylation percentage, are not deep-seated, are resected and undergo Stupp dose radiotherapy and TMZ (Table 2), supporting prior research.^19–22^ It was also related to having a later post-radiotherapy MRI.

### Nonimaging Models

**Figure 2.**
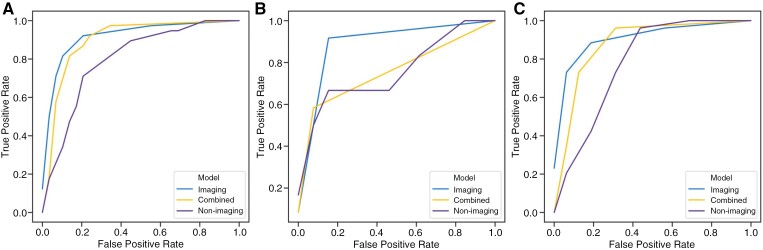
Receiver-operating characteristic curves for imaging, combined, and nonimaging models on holdout test data. (a) Model performances on the amalgamated test set. AUCs were 0.93, 0.91, and 0.79 for the imaging, combined, and nonimaging models respectively. (b) Model performances on the retrospective test set. AUCs were 0.92, 0.94, and 0.76 for the imaging, combined, and nonimaging models respectively. (c) Model performances on the external, prospective test set. AUCs were 0.93, 0.89, and 0.78 for the imaging, combined, and nonimaging models respectively. *AUC*: area under the receiver-operating characteristic curves.

Among all nonimaging machine learning models, logistic regression with reduced features was selected as the optimal classifier based on the highest validation AUC. The optimal logistic regression model had regularization parameter (C) set to 1.0 and 10 features retained (male sex, methylated MGMT status, unmethylated MGMT status, unknown MGMT status, initial biopsy, initial resection, standard radiotherapy dose, reduced radiotherapy dose, reduced TMZ dose, and no TMZ). These were all one-hot encoded categoric variables (for example, separate variables encoded if a patient had methylated, unmethylated, or unknown MGMT status). The AUCs for retrospective, prospective and amalgamated test sets were 0.76, 0.78 and 0.79, respectively (Table 3); performances did not differ between test sets (all *P* > .05). To aid with assessments of model performances and generalizability across test sets, Figure 2 shows receiver-operating characteristic (ROC) curves for all models (imaging, combined, and nonimaging) on the amalgamated, retrospective, and prospective test sets.

**Table 3.** Holdout Test Set Performances from Imaging, Combined (Imaging/Nonimaging), and Nonimaging Models. The Retrospective Test Set is an Internal Validation Dataset. The Prospective Test Set is an External Validation Dataset Using Data from Geographically Distinct Sites. The Amalgamated Test Set Refers to the combination of the retrospective and prospective test sets

Description	AUC^a^	Precision	Recall	F1	Specificity	NPV^b^	BAR^c^	Accuracy
*Amalgamated Test Set (n = 48 Patients, from 10 Centers)*								
**Imaging model**	**0.93 ± 0.07** ^*^	**0.77**	**0.89**	**0.83**	**0.83**	**0.92**	**0.86**	**0.85**
Combined model	0.91 ± 0.08	0.63	1.00	0.78	0.62	1.00	0.81	0.77
Non-imaging model	0.79 ± 0.12	0.67	0.32	0.43	0.90	0.67	0.61	0.67
*Retrospective Test Set (n = 19 Patients, from 2 Centers)*								
Imaging model	0.92 ± 0.12	0.67	1.00	0.80	0.77	1.00	0.88	0.84
**Combined model**	**0.94 ± 0.11**	**0.55**	**1.00**	**0.71**	**0.62**	**1.00**	**0.81**	**0.74**
Nonimaging model	0.76 ± 0.19	0.67	0.33	0.44	0.92	0.75	0.62	0.74
*Prospective Test Set (n = 29 Patients, from 8 Centers)*								
**Imaging model**	** 0.93 ± 0.09** ^*^	**0.85**	**0.85**	**0.85**	**0.88**	**0.88**	**0.86**	**0.86**
Combined model	0.89 ± 0.11	0.68	1.00	0.81	0.63	1.00	0.81	0.79
Nonimaging model	0.78 ± 0.15	0.57	0.31	0.4	0.81	0.59	0.56	0.59

^a^ *AUC*: area under the receiver-operating characteristic curve. The key results for machine learning models are the generalizability of holdout test set values. We also compute the sample size-based 95% CI using the Bernoulli trials formula $\left(z\times \sqrt{\frac{AUC\times \left(1-AUC\right)}{n}}\right)$.

^b^ *NPV*: negative predictive value.

^c^ *BAR*: balanced accuracy rate.

^*^Significantly different AUC compared to the nonimaging model using DeLong’s test with a threshold of *P* ≤ .05.

Bold rows are those with the highest AUC scores.

### Imaging and Combined Models

The parameters used to optimize the imaging model are shown in Supplementary Appendix F1. Initializing the imaging model with pretrained weights from 10 000s of brain MRIs^25,26^ improved performance considerably (with and without pretraining on amalgamated test set gave AUCs of 0.93 and 0.64 respectively; *P* = .003). Therefore, performances of imaging (and combined) models initialized with pretrained weights are reported (Table 3). The imaging model AUCs for retrospective, prospective and amalgamated test sets were 0.92, 0.93 and 0.93, respectively, and did not differ in performance between sets (*P* > .05) (Figure 2).

For the combined model, AUCs for retrospective, prospective and amalgamated test sets were 0.94, 0.89 and 0.91, respectively; performances did not differ across test sets (*P* > .05).

All models applied a survival classification threshold of 0.50; an analysis of decision threshold selection is presented in Supplementary Appendix F2. A description of the interval between radiotherapy completion and the first post-radiotherapy MRI study for patients in the amalgamated test set is presented in Supplementary Appendix G1.

### Model Comparison

One way for imaging-based biomarkers to be valuable in the clinic is that, when compared to freely-available nonimaging biomarkers, there is an incremental increase in predictive accuracy when biomarkers are combined. An incremental increase in performance was not clearly proven for the combined model. We found that whilst there was a trend for enhanced performance in the amalgamated test set (AUC 0.91 vs 0.79, *P* = .07), in retrospective and prospective test sets this was less clear (Figure 2) (*P = *.11 and *P* = .16).

Another, plausibly optimal, way for imaging-based biomarkers to be valuable clinically is that, when compared to freely-available nonimaging biomarkers, the predictive accuracy is higher. The advantage of using an imaging model alone is that it can be applied in isolation, without needing additional information gathering. The imaging model outperformed the nonimaging model in amalgamated and prospective test sets (AUC, *P* ≤ .05) (Table 3 and Figure 2). However, performances did not significantly differ on the retrospective test set (*P = *.14); a comparison of receiver-operating characteristic curves suggests that this may be related to the smaller retrospective test set size (retrospective test *n* = 19) (Appendix G2). The combined model was not superior to the imaging model in any test set (*P* > .05), despite the combined model incorporating information on the interval between radiotherapy completion and follow-up imaging (the interval was different in the 2 groups). To further assess whether the model could complement evaluations made in routine hospital settings, we performed a comparison against expert clinical raters reported in Supplementary Appendix H.

### Imaging Model Explainability

**Figure 3.**
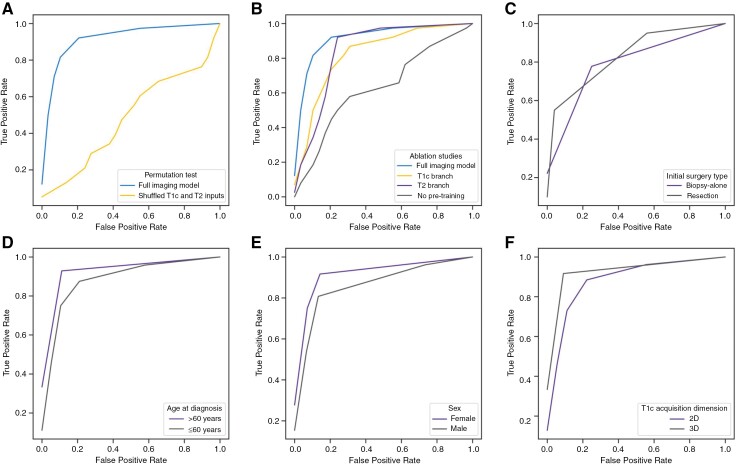
Receiver-operating characteristic curves displaying imaging model performances for additional analyses run on the amalgamated test set. (a) Permutation test results (full imaging model, AUC = 0.93; permutation test, AUC = 0.49*). (b) Results from ablation studies (full imaging model, AUC = 0.93; predictions from T1c branch, AUC = 0.83*; predictions from T2 branch, AUC = 0.85; trained model initializing random weights—ie, with no pretraining, AUC = 0.64*). Panels (c–f) show imaging model results disaggregated for sample subgroups. (c) Performances based on the initial surgery type (biopsy-alone, AUC = 0.89; resection, AUC = 0.87). (d) Curves plotted separately for age at first diagnosis (>60 y, AUC = 0.98; ≤60 y, AUC = 0.89). (e) Performances based on sex (female, AUC = 0.96; male = 0.89). (f) Performances split by the acquisition dimension of the input T1c MRI (2D, AUC = 0.90; 3D, AUC = 0.98). *AUC*: area under the receiver-operating characteristic curves. *T1c*: contrast-enhanced T1-weighted MRI. *T2*: T2-weighted MRI. *: significantly different AUC compared to the full imaging model using DeLong’s test with a threshold of *P *≤ .05.

Based on the findings that available nonimaging features did not improve predictive performances, and that the combined model was not superior to the imaging model, the imaging model was selected over nonimaging and combined counterparts for further analysis. ROC curves showing results from the permutation test and ablation studies are provided in Figure 3. Model performances are plotted separately for sample subgroups (initial surgery type, age group, sex, and T1c acquisition dimension; Figure 3). Further detail on imaging model results from the permutation test and ablation studies, along with performances disaggregated for sample subgroups is provided in Supplementary Appendix G2. The permutation test AUC of 0.49 indicates that the model was not performing by chance.

Ablation studies showed that test set performance using the merged branch was similar to using the T2 branch alone (comparison of AUCs across amalgamated test set, *P* = .19), but better than the T1c branch alone (*P* = .048). Performances were similar when using only 1 sequence (T1c versus T2 branches, *P = *.41). Together, this suggests that on the rare occasion that a patient does not receive gadolinium (for example, due to high-grade renal failure, or patient refusal), predictions may remain accurate with only the T2 sequence. We found that test set performance dropped considerably when not training with transfer learning, where initial weights were pretrained on a brain MRI dataset ×100 larger than the training dataset (AUCs with and without pretraining 0.93 and 0.64, respectively; *P* = .003). This shows that medical image classifiers with high-dimensional and high-resolution inputs such as brain MRIs may benefit from pretraining on larger datasets.

**Figure 4.**
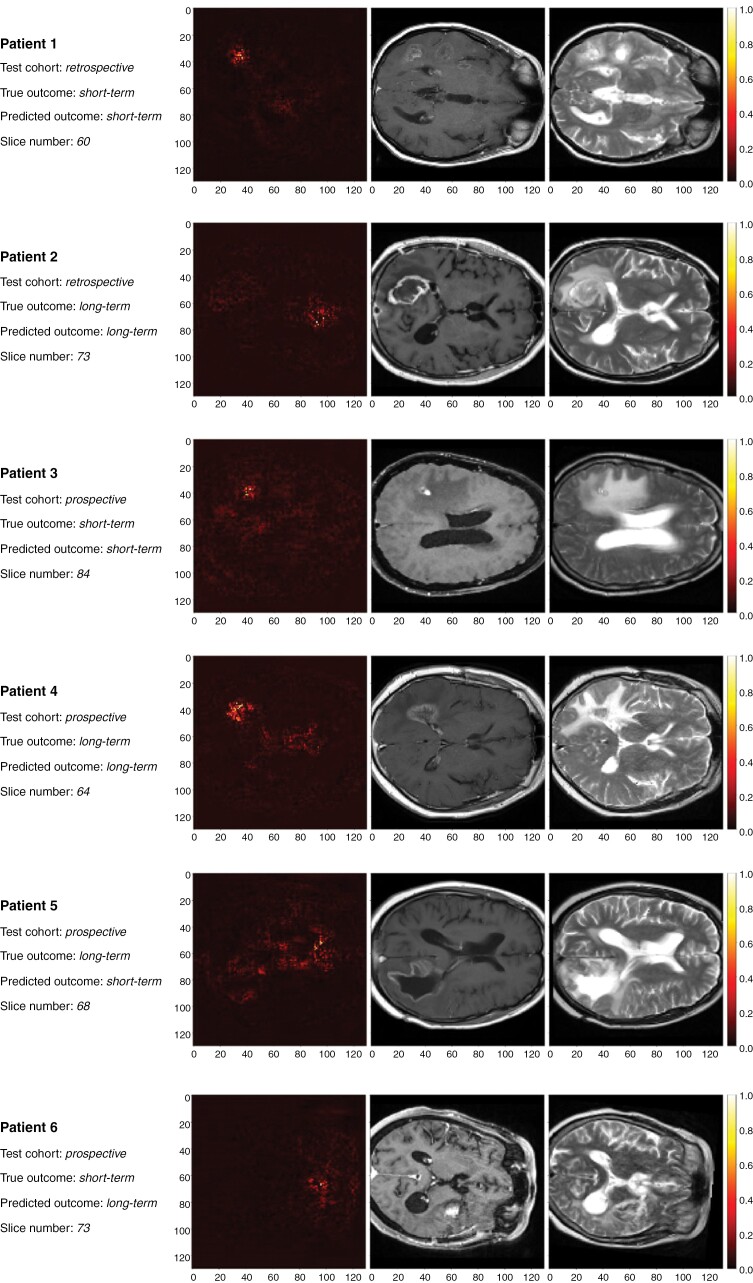
Saliency maps from guided backpropagation on the merged branch of imaging models using T1c and T2 inputs. Patients from retrospective and prospective test sets were selected including erroneous classification predictions (patients 5 and 6). *T1c*: contrast-enhanced *T*_1_—weighted MR sequence. *T2*: *T*_2_—weighted MR sequence.

Saliency maps based on predicted survival outcome from the imaging model are presented in Figure 4. These show examples of short-term and long-term survivors from retrospective and prospective external test sets, along with erroneous predictions of both survival outcomes. Across patients, there appears to be variation in the location, size and number of brain areas that are salient. For example, some maps seemingly display coarse localization of tumor regions, as well as ventricles. It is plausible that it may be more difficult to interpret appearances associated with long-term compared to short-term survival in MRIs and saliency maps (ie, to identify the absence of expected deterioration). Nonetheless, we can make some tentative observations. Patient 2, for example, was correctly predicted to have subsequent long-term survival. In this case, the presented slices suggest a relatively greater contribution from ventricular areas than the treated tumor region. This suggests that both tumor and nontumor regions provide informative features for deep learning models, and jointly contribute to survival predictions. Among misclassified patients, it is conceivable that model weights associate ventriculomegaly with short-term survival (for example, patient 5). Further analysis of saliency maps is presented in Supplementary Appendix I. However, it should be noted that saliency maps alone do not identify features that are easily interpretable to human readers.^32^

## Discussion

We present the first known model that uses imaging to distinguish short-term and long-term survivors within 8 months of completing radiotherapy. Eight months represents the period of time to complete adjuvant chemotherapy. Using a multicenter cohort, we built a model with T1c and T2 inputs. The transfer learning approach improved predictions. There was no clear benefit of generating predictions with nonimaging data. Using the T2 scan alone was not inferior to using both sequences. The imaging model seemed to generalize both to retrospective and external, prospective test data.

One strength of this study is providing insight into the extent to which neural networks predicting post-treatment survival generalized across multiple external centers. External, prospective sites showed a higher proportion of 2D scanning and short-term survivors than retrospective data which may have been a potential source of bias. Therefore, we stratified data to allow better evaluation of predictions on short-term and long-term survivors, and both 2D and 3D acquisitions. Based on similar performances across the retrospective and external prospective datasets, the imaging model may be robust to variations in imaging protocols and class imbalances.

Another key contribution is the finding that transfer learning can offer a strong benefit to models with large numbers of parameters and small training samples. This accords with other research evaluating the benefits of transfer learning for MRIs of glioma patients. For example, 1 study combining low-grade and high-grade gliomas found that pretraining improved the classification accuracy of a deep learning radiogenomic model.^33^ Another study combined classical radiomics features with those extracted from a pretrained neural network to predict overall survival of glioblastoma.^34^ These studies used natural images for pretraining and predicted outcomes from cropped 2D slices of tumor regions from preoperative MRIs. In comparison, the model used for pretraining in our study was trained on thousands of brain MRIs and was highly successful at detecting abnormalities.^25,26^

Previous research that successfully applied machine learning to predict survival of glioblastoma has largely focused on pretreatment timepoints. One study used a DenseNet-based network with multiple branches to predict 3-year survival from 2D T1c and T2 slices.^4^ Another applied a neural network to quantify the temporalis muscle; this predicted survival in distinct datasets.^5^ Several studies with multicenter data extracted radiomics features from preoperative tumor segmentations and applied machine learning to predict survival.^3^ To our knowledge, prior studies have not demonstrated the benefits of classical or deep machine learning methods on predicting outcomes from post-treatment time points, and with whole-brain inputs requiring minimal preprocessing.

Our imaging model is a contribution towards developing networks that could be applied to aid decision-making in hospitals. The 2-year survival rate of glioblastoma is just 18%.^35^ Such models could prompt closer MRI surveillance of suspected short-term survivors, compared with patients expected to show initial treatment response. Large prospective studies replicating high predictive performances in clinical settings are now desirable. If validated, studies assessing improvements to patient management are required. Researchers could also investigate extending model applicability using, for example, curated second-line therapy trial datasets.

Our model predicted post-radiotherapy *survival* using imaging as a prognostic biomarker which can be used to stratify patients into those requiring early second-line treatment or trial enrollment. An alternative model might predict tumor *treatment response* using imaging as a monitoring biomarker.^6^ While not the focus of our study which incorporates all patients consecutively (including complete response, partial response, stable disease, progression, and pseudoprogression), interpreting post-radiotherapy structural MRIs in clinical settings is typically challenging due to difficulty in distinguishing recurrent disease from treatment-related effects—particularly for pseudoprogression.^2,3,6,11,17,36–38^ However, labelling progression—and pseudoprogression—requires the availability of repeated T1c imaging obtained in a timely manner per patient, accompanied by accurate measurements of bidirectional diameters of contrast-enhancing tumors.^37,38^ Prior research has reported that there can be substantial inter-rater variability in these measurements, however, which can confound evaluations of treatment response.^39–41^ One reason for measurement variability is the irregular shape at the tumor margin,^3^ while another relates to similarities in signal intensity between tumor and nontumor if precontrast T1c scans are not studied carefully.^42^ To rule out factors that potentially confound assessments, data on prescribed steroids and longitudinal patient symptom profiles are additionally needed. In contrast, the approach presented here uses overall survival as the reference standard, free from inter-rater variability and requirements for RANO-compliant longitudinal data collection. Our study was not designed to identify the first occurrence of true tumor progression (and thereby rule out pseudoprogression, which is expected to be associated with longer survival). However, our approach has the potential to provide all tumor boards monitoring patients at all time periods after radiotherapy with an accurate *a priori* survival prediction gained at the first post-radiotherapy scan, thereby improving management decision confidence, including for example, the challenging scenario of pseudoprogression.

While predictions did not improve when incorporating nonimaging features, we had a limited number of these variables. Combined models with a greater range of tumor-related data might show better performances (eg, Ki67 percentage, ATRX status, genomic variables). Models could also integrate earlier MRI studies which may contain useful features for improving prognostic predictions, for example presurgical and preradiotherapy studies. For now, a model that could translate most easily across centers would likely benefit from a pragmatic approach that requires collecting widely available nonimaging features and cross-sectional (rather than longitudinal) imaging.

A potential limitation is that we did not consider other MRI sequences that may provide insights into tumor recurrence (eg, diffusion or perfusion imaging).^43^ However, our models used T1c and T2 sequences to maximize clinical utility and translation across hospitals. These sequences were consistently acquired at all centers; conversely, more advanced MRIs are less commonly available.^17,18^ Incorporating other anatomical sequences desirable for brain tumor imaging, such as FLAIR sequences, was not also pursued as it would have reduced the patient cohort in this UK-based study where FLAIR imaging was not always performed. A downstream constraint of building a model without the most common MRI sequences is that it reduces the potential for clinical translation. Nonetheless, future models could investigate the extent to which models built with alternative imaging protocols (for example, advanced imaging as well as FLAIR) can predict post-treatment survival.

Another limitation is that we used a small dataset whereas DenseNet^29^ is a large model and whole-brain images provide many inputs per patient. Beyond pretraining, future research could use smaller inputs, eg, bounding boxes cropped to initial tumor sites. This was not pursued because (i) extracranial information is linked with overall survival^5^; (ii) contrast-enhancing masses remote to the initial site signal recurrence (and shorter survival); (iii) data preprocessing that aligned with pretraining preprocessing was favored^25^; and (iv) whole-brain images require minimal preprocessing (plausibly reducing barriers to translation).

In this multicenter study, we developed a model that predicts survival within 8 months of completing radiotherapy. The model is intended for use for patients undergoing optimal treatment as well as the under-studied cohort of patients undergoing modified treatments. A neural network with T1c and T2 branches showed generalizable classification on both retrospective and external, prospective test cohorts. If validated in large prospective studies, such approaches could be used to distinguish patients who show an initial response to radiotherapy from those requiring closer image-based monitoring and second-line treatments (or termination of ineffective treatment).

## Supplementary Material

noae017_suppl_Supplementary_Appendix

## Data Availability

Data generated or analyzed during the study are available from the corresponding author by request.
