# Supplementary material for: Glioblastoma and radiotherapy: A multicenter AI study for Survival Predictions from MRI (GRASP study)
Source: Neuro Oncol. 2024 Jan 29;26(6):1138–51. doi: 10.1093/neuonc/noae017 (PMC11145448; doi:10.1093/neuonc/noae017)
Supplement: noae017_suppl_Supplementary_Appendix [file noae017_suppl_supplementary_appendix.docx]

**Supplementary Material**

**Appendix A. Overview of the treatment and imaging pathway for glioblastoma**


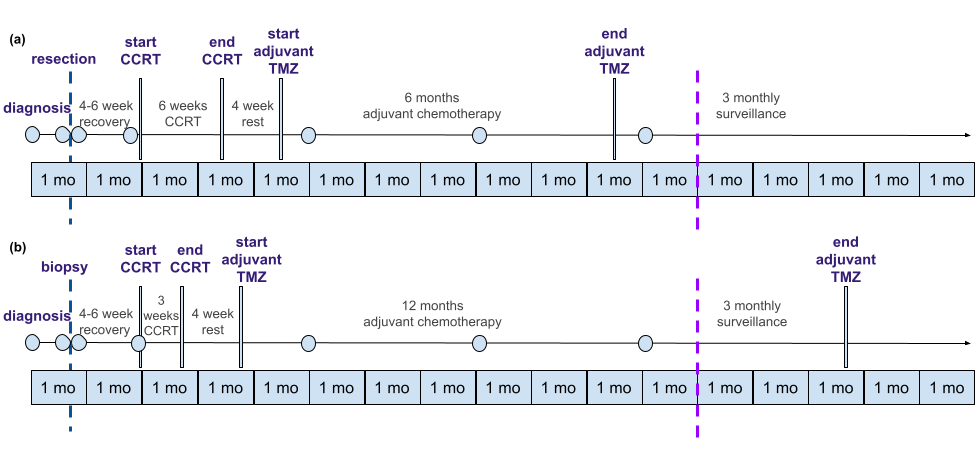


**Figure A1.** Simplified illustration of the recommended treatment and imaging pathway for glioblastoma. Some patients may not tolerate recommended post-surgical therapies, and therefore undergo an altered course of treatment. Circles are illustrative of recommended interval imaging timepoints. To inform patient management, MRIs are often performed after initial surgery, during radiotherapy planning, and at three-monthly intervals (or if clinically deteriorating) during follow-up after radiotherapy completion^1,2,3,4^. **(a)** Pathway for patients receiving the optimal treatment (i.e., completing the “Stupp” protocol, which consists of surgical resection, followed by radiotherapy with concomitant TMZ, then adjuvant TMZ)^5,6^. **(b)** Pathway for patients receiving a common modified treatment. Modified treatment, as shown in this example, often includes a shorter course of CCRT with a lower dose of radiotherapy; a longer course of adjuvant chemotherapy may be prescribed.

*CCRT*: radiotherapy and concomitant chemotherapy. *TMZ*: temozolomide.

**Appendix B. Patient and Public Feedback**

The research proposal was presented to the Next Generation Medical Imaging Advisory Group at King’s College London (January 2021), and the Guy’s Cancer Group at Guy's and St Thomas' Hospital (February 2021).

Based on feedback, this study considered (i) the influence of non-imaging features on model development, and (ii) in addition to patients undergoing optimal treatment (i.e., completing the “Stupp” protocol, which consists of surgical resection, followed by radiotherapy with concomitant TMZ, then adjuvant TMZ), inclusion of patients without optimal treatment (e.g., those who could not tolerate full-dose of radiotherapy or temozolomide chemotherapy, or who had an initial biopsy without a maximal safe resection). Test evaluation focused on the area under the receiver operating characteristic curve (AUC) metric. Groups commented that multi-center, prospective test data would provide greater reassurance of detecting post-treatment changes in an evidence-based manner.

**Appendix C. Patient Cohort**


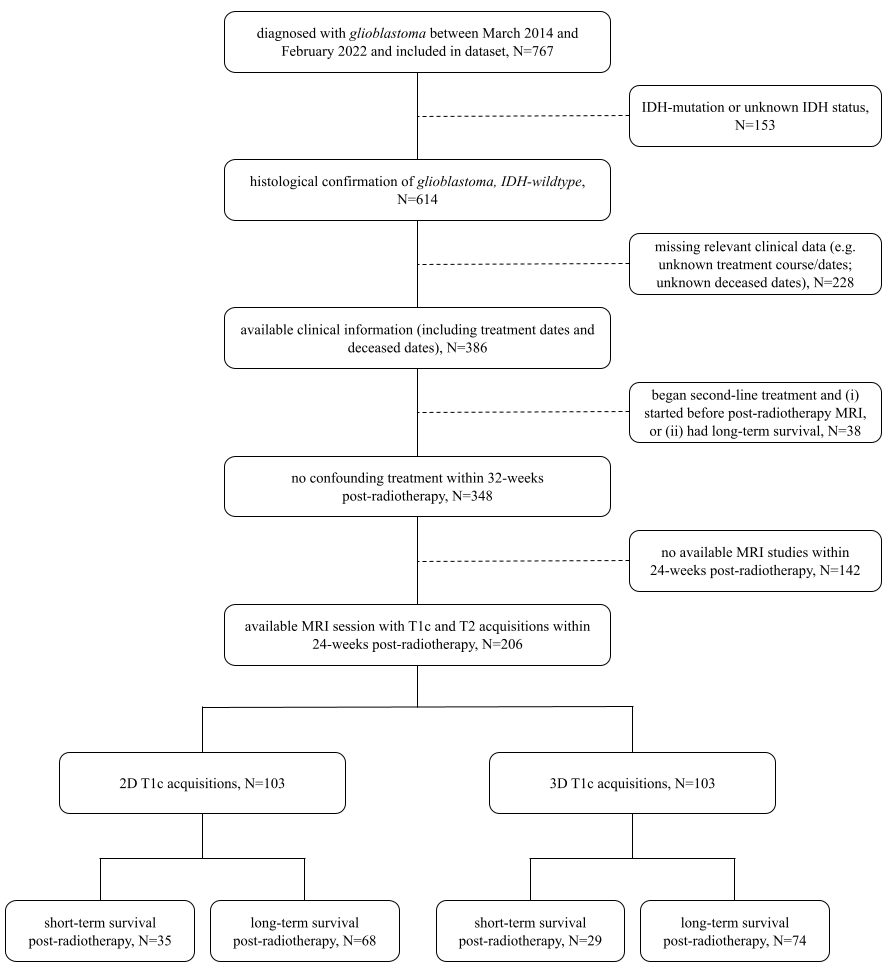


CONSORT diagram displaying flow of patients included in analyses. Also demonstrated is the different contrast-enhanced *T*_1_-weighted (T1c) sequences categorized as 3D or 2D.

**Appendix D. Dataset sampling**

**Appendix D1.** Patient characteristics in full and test datasets.

To check for potential bias(es) in the overall dataset sampling strategy, we compared the portion of patients in the test set to the full dataset and measured the sampling error for the following variables: survival outcome, contrast-enhanced *T*_1_-weighted acquisition dimension (2D or 3D), initial surgery type (biopsy or maximal safe resection), MGMT methylation status at first diagnosis, and age at first diagnosis. These findings are presented in Table B1 below. Sampling for validation folds stratified outcome, acquisition dimension, and acquisition center (KCH/LTHT/UCLH) in training data. Surgery type, MGMT methylation status, and age are reported here as these factors are related to survival of glioblastoma^7-10^; these were not stratified during cross-validation due to low patient numbers after stratifying for three variables.

**Table D1.** Patient characteristics of the full dataset compared to test data described by survival outcome, MRI acquisition dimension, surgery type, MGMT status, and age group. Test set sampling error is relative to the full dataset.

| **Stratified**  **variable** | | **All patients, N=206** | **Combined**  **test set,**  **N=48** | | **Retrospective**  **test set,**  **N=19** | | **Prospective**  **test set,**  **N=29** | |
| --- | --- | --- | --- | --- | --- | --- | --- | --- |
|  |  | **N (%)** | **N (%)** | **Sampling error** | **N (%)** | **Sampling error** | **N (%)** | **Sampling error** |
| Survival outcome | Short-term | 64  (31.1%) | 19 (39.6%) | 27.4 | 6  (31.6%) | 1.64 | 13 (44.8%) | 44.29 |
|  | Long-term | 142  (68.9%) | 29  (60.4%) | -12.4 | 13 (68.4%) | -0.74 | 16 (55.2%) | -19.96 |
| Acquisition dimension | 2D | 103  (50.0%) | 31 (64.6%) | 29.17 | 9 (47.4%) | -5.26 | 22 (75.9%) | 51.72 |
|  | 3D | 103  (50.0%) | 17  (35.4%) | -29.17 | 10 (52.6%) | 5.26 | 7 (24.1%) | -51.72 |
| Surgery  type | Biopsy | 48  (23.3%) | 13  (27.1%) | 16.23 | 4  (21.1%) | -9.65 | 9  (31.0%) | 33.19 |
|  | Resection | 158  (76.7%) | 35 (72.9%) | -4.93 | 15  (78.9%) | 2.93 | 20 (69.0%) | -10.08 |
| MGMT status | Methylated | 87  (42.2%) | 20  (41.6%) | -1.34 | 8  (42.1%) | -0.30 | 12  (41.4%) | -2.02 |
|  | Unmethylated | 114  (55.3%) | 28  (58.3%) | 5.41 | 11  (57.9%) | 4.62 | 17  (58.6%) | 5.93 |
|  | Unknown | 5  (2.4%) | 0  (0.0%) | - | 0  (0.0%) | - | 0  (0.0%) | - |
| Age at first diagnosis | ≤ 60 years | 89  (43.2%) | 32  (66.7%) | 17.38 | 14  (73.7%) | 29.73 | 18  (62.1%) | 9.28 |
|  | > 60 years | 117  (56.8%) | 16  (33.3%) | -22.85 | 5  (26.3%) | -39.09 | 11  (37.9%) | -12.20 |

**Appendix D2**

A visualization of the distribution of time between radiotherapy completion and the first MR study (used as inputs to imaging/combined models) is presented in Figure B1, split by survival outcome.  The study was pragmatic, and imaging was carried out in line with local practice, and at a time dictated by clinical protocols, or additional clinical concerns. Detail on local UK imaging protocols is shown in the GIN CUP study^11^.


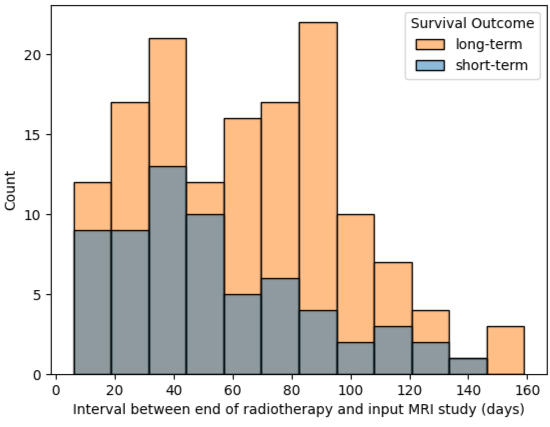


**Figure D1**. Histogram showing time between end of radiotherapy and the first MRI examination for all patients, stratified by survival outcome. The first MRI images after radiotherapy completion are used as inputs for the imaging model, as well as the duration between end of radiotherapy and the scan.

**Appendix E. Further information on survival classifiers**

**Appendix E1**. Description of non-imaging models

*Description of non-imaging classical machine learning models*

Four types of machine learning models were applied to training data with parameter tuning. Logistic regression models, linear support vector classifiers (SVCs) and gaussian SVCs were run with tuning of the regularization parameter (C) (range of C: 0.1 to 1000). For the gaussian SVC, gamma coefficients were additionally tuned using grid search (range of gamma: 0.1 to 100). Finally, decision tree classifiers were developed with selection between gini and entropy criteria for evaluating partitions. Based on validation performances, backward sequential feature selection was applied until the area under the receiver-operating characteristic curve (AUC) decreased.

Numeric variables were standardized to unit variance using training data, and categoric variables were one-hot encoded. Where data were missing, three approaches were used: (i) mean/mode imputation was used with labels added identifying imputed inputs, (ii) patients with missing data were excluded, (iii) variables with missing data were excluded.

*Description of non-imaging fully-connected neural networks*

As an additional baseline measure, we trained shallow neural networks to predict survival outcomes from available non-imaging features alone (demographic, histologic, tumor-related, and prior treatment variables). These non-imaging features were passed through either one or two fully-connected linear layers before providing the binary survival prediction. Tuned parameters were the number of linear layers, learning rate and schedule, and probability of dropout; where there were two linear layers, the size of linear layers was also tuned (range=4-24). The fully-connected neural networks did not have a higher validation performance than corresponding machine learning models, so were not pursued further. Models were developed with PyTorch^12^.

**Appendix E2.** Description of alternative imaging model.

Instead of splitting contrast-enhanced T1-weighted (T1c) and T2-weighted (T2) MR sequence inputs across branches, we also tested a version of the model with both sequences joined as two channels (one branch). Pretrained weights were combined by either taking the average weight per convolutional layer and block, or by selecting the maximum weight. These models were not pursued further as they displayed poorer performances than counterparts with separate branches per input MR sequence.


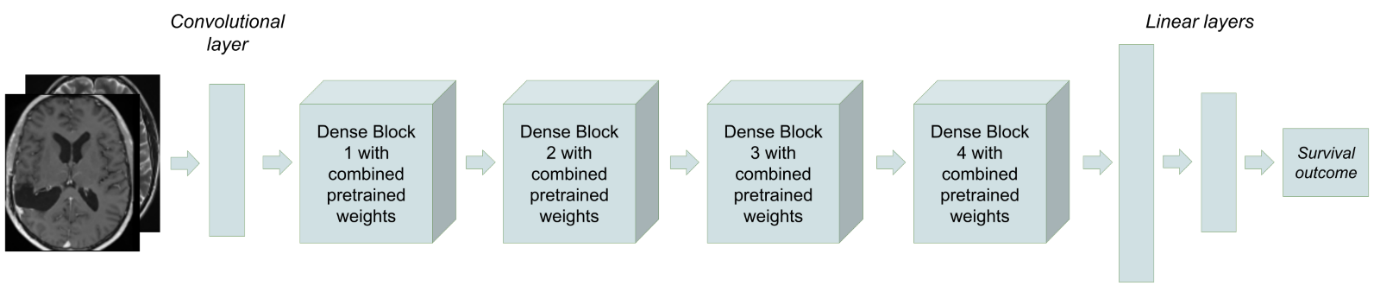


**Appendix E3.** Procedure for training and tuning imaging and combined models.

Models were trained measuring cross-entropy loss weighted by class, and with the Novograd optimizer. Tuned (hyper-)parameters included: learning rate and schedule, linear layer sizes, number frozen/updated pretrained blocks, loss weighting per branch, dropout, pooling, and degree/probability of augmentation.

**Appendix F.**

**Appendix F1. Parameters for the optimal imaging model**

After tuning the imaging model, pretrained weights were frozen for the first convolutional layer and two dense blocks per branch. Blocks were flattened via maximum pooling; flattened feature vectors were mapped to a 1x56 then 1x2 vector per branch; the SeLU activation function was used. The merged branch therefore inputs a 1x112 vector. Losses were weighted for T1c, T2, and merged branches at a ratio of 1:1:3 respectively. The probability for each augmentation was 0.65. Applied augmentations included random left-right flipping, zooming, shearing, translation, rotation, adjusting intensity, adjusting contrast, adding Gaussian noise, and adding coarse dropout. A cyclical learning rate was applied (range: 4^-8^-2^-5^). The model was trained for 170 epochs before frozen and evaluated on holdout test data.

**Appendix F2. Selecting the classification threshold**

**Description of threshold analysis**

All models applied a classification threshold of 0.50 to determine the survival prediction label.

As an additional analysis to investigate the optimal decision threshold, Youden’s J statistic was calculated on validation folds for the imaging model; the mean threshold was selected.

**Test set results when applying Youden’s J threshold**

The Youden’s J analysis suggested a threshold of 0.35. Applying this decision threshold to test set predictions did not improve imaging model performances (AUC=0.75; balanced accuracy rate=0.74). Therefore, a threshold of 0.50 was retained for all models (imaging/combined/non-imaging).

**Appendix G. Additional analyses of test set predictions**

**Appendix G1. Interval between radiotherapy completion and MRI study used for survival prediction**

As a supplementary analysis, we investigated whether survival predictions may be influenced by the interval between radiotherapy completion and the MRI study used as model inputs. For potential clinical translation, it is important to assess if there is a potential source of bias, where patients with a longer duration between radiotherapy completion and the first MRI may be predicted as long-term survivors and vice versa. For example, rather than identifying features related to future survival in neuroimaging, the model may be identifying that patients who are less well are brought in for MRI follow-up sooner than those who are responding to treatment. This possibility remains despite model inputs being limited to those MRIs obtained within 24-weeks of radiotherapy completion. We therefore further investigated patients with erroneous survival predictions.

The amalgamated test set has n=48 patients, of whom 19 were short-term survivors (39.6%). The imaging model made erroneous predictions for 7/48 patients (85.4% accuracy). The interval between radiotherapy completion and the MRI study used as model inputs is shown for the test set, based on the survival outcome:


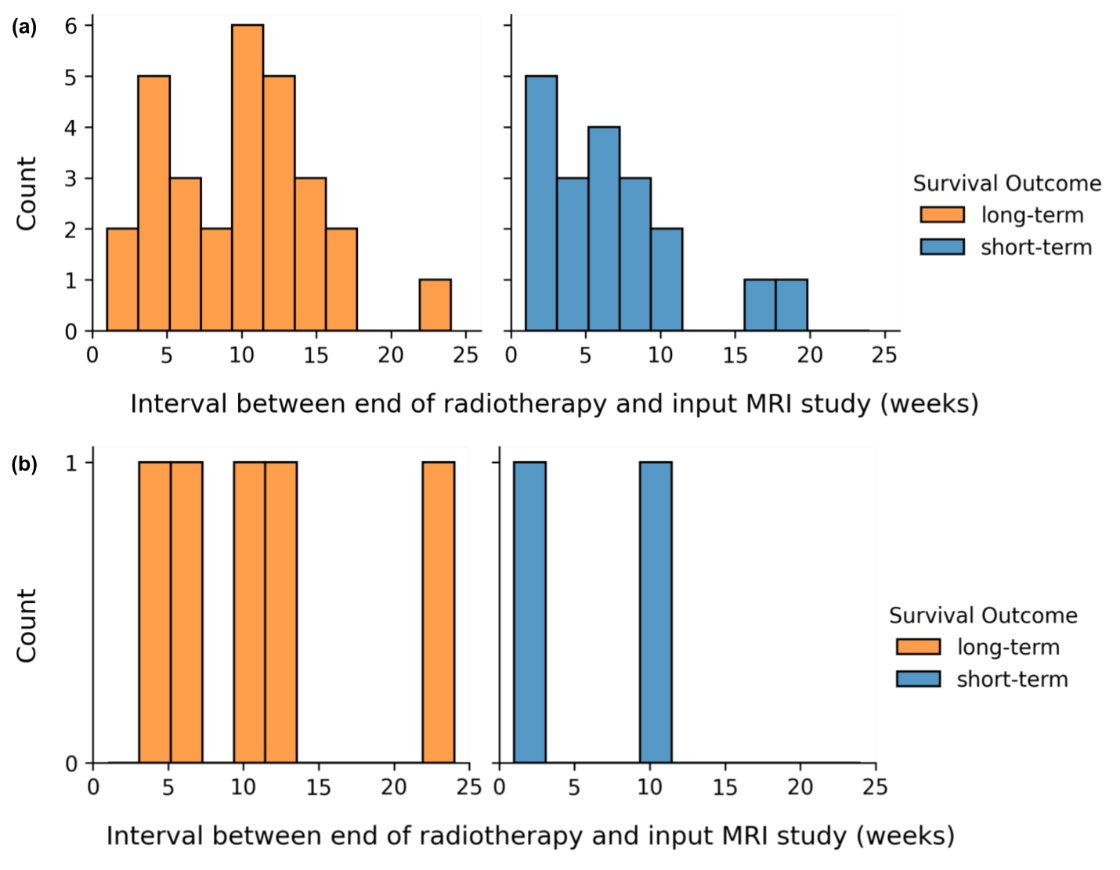


**Figure G1.** Interval between radiotherapy completion and the MRI study used as imaging (and combined) model inputs. **(a)** The interval for the amalgamated test set, split by survival outcome. **(b)** Intervals for patients with erroneous prognostic predictions (where long-term survivors were misclassified as short-term survivors, and vice versa).

Importantly, we found that the combined model did not perform significantly better than the imaging model, despite receiving this interval as an input variable. Furthermore, based on the overlapping intervals between misclassified short-term and long-term survivors in panel (b) of Figure G1, it is not overtly evident that the imaging model is biased due to variations in the duration between radiotherapy completion and the first post-radiotherapy MRI study. Further research should verify this expectation with a larger test set however, particularly for patients with the first MRI study obtained between 20-24 weeks after radiotherapy completion.

**Appendix G2. Imaging model performances for additional analyses run on the amalgamated test set**

Performance of the imaging model on the amalgamated test set is further described in Table G2. Additional analyses were performed including permutation testing, ablation studies, and grouping predictions by sample characteristics.

**Table G2.** Imaging model performances for additional analyses run on the amalgamated test set (the amalgamation of the retrospective and prospective test sets). Performances are shown for the permutation test and ablation studies; they are also reported disaggregated for sample subgroups (surgery type, age (>60years), sex, and acquisition dimension).

| **Description** | **AUC*^a^*** | **Precision** | **Recall** | **F1** | **Specificity** | **NPV*^b^*** | **BAR*^c^*** | **Accuracy** |  |
| --- | --- | --- | --- | --- | --- | --- | --- | --- | --- |
| *Full imaging model* | | | | | | | | | |
| Imaging model | 0.93 | 0.77 | 0.89 | 0.83 | 0.83 | 0.92 | 0.86 | 0.85 |  |
| *Permutation test results* | | | | | | | |  | |
| Shuffled T1c*^d^* and T2*^e^* inputs | 0.49* | 0.40 | 1.00 | 0.57 | 0.00 | 0.00 | 0.50 | 0.40 |  |
| *Ablation studies* | | | | | | | | | |
| Predictions from T1c branch | 0.83* | 0.65 | 0.89 | 0.76 | 0.69 | 0.91 | 0.79 | 0.77 |  |
| Predictions from T2 branch | 0.85 | 0.67 | 0.63 | 0.65 | 0.79 | 0.77 | 0.71 | 0.73 |  |
| Train model initializing random weights | 0.64* | 0.50 | 0.21 | 0.30 | 0.86 | 0.63 | 0.54 | 0.60 |  |
| *Initial surgery type (n)* | | | | | | | |  | |
| Biopsy-alone (13) | 0.89 | 0.89 | 0.89 | 0.89 | 0.75 | 0.75 | 0.82 | 0.85 |  |
| Resection (35) | 0.87 | 0.69 | 0.90 | 0.78 | 0.84 | 0.95 | 0.87 | 0.86 |  |
| *Age at diagnosis (n) (missing n=1)* | | | | | | | |  | |
| >60 years (16) | 0.98 | 0.78 | 1.00 | 0.88 | 0.78 | 1.00 | 0.89 | 0.88 |  |
| ≤60 years (31) | 0.89 | 0.77 | 0.83 | 0.80 | 0.84 | 0.89 | 0.84 | 0.84 |  |
| *Sex (n)* | | | | | | | |  | |
| Female (20) | 0.96 | 0.71 | 0.83 | 0.77 | 0.86 | 0.92 | 0.85 | 0.85 |  |
| Male (28) | 0.89 | 0.80 | 0.92 | 0.86 | 0.80 | 0.92 | 0.86 | 0.86 |  |
| *T1c acquisition dimension (n)* | | | | | | | | | |
| 2D (31) | 0.90 | 0.79 | 0.85 | 0.81 | 0.83 | 0.88 | 0.84 | 0.84 |  |
| 3D (17) | 0.98 | 0.75 | 1.00 | 0.86 | 0.82 | 1.00 | 0.91 | 0.88 |  |

*^a^ AUC*: area under the receiver operating characteristic curve.

*^b^ NPV*: negative predictive value.

*^c^ BAR*: balanced accuracy rate.

*^d^ T1c*: contrast-enhanced T1-weighted MRI.

*^e^ T2*: T2-weighted MRI.

* : significantly different AUC compared to the full imaging model using DeLong’s test with a threshold of *p*≤0.05

**Appendix H. Comparative predictions from expert clinical raters**

In the main manuscript, we provided a comparison of predictions from artificial intelligence models based on imaging-alone, combined information, and non-imaging clinical variables alone. The imaging model was selected for further analysis, based on the observation that using non-imaging features did not improve model performances. Readers may also be interested in how the selected model performs in comparison to physicians’ interpretation of the same imaging data. It is acknowledged that such a prediction of long- or short-term survival is not expected when reporting on neuroimaging in clinical settings. Nonetheless, such a comparison might help to determine whether the model could be complementary in routine hospital practice.

To provide this comparison, we conducted a blinded study with predictions obtained from expert clinicians reviewing patient imaging. Three senior neuroradiologists (UK consultant grade; US attending equivalent) who present the imaging at the joint neuro-oncology meeting (UK multi-disciplinary meeting; US tumor board) at three UK neuro-oncology centers, made the equivalent survival predictions as the image-based model presented here, using the same T1 post-contrast (T1c) and T2 MRIs.

Predictive performance of each rater on the amalgamated test set is presented in Table H1, in addition to performance based on inter-rater consensus (i.e., the mode/majority vote). Since raters were predicting the binary survival outcome rather than providing prediction probabilities per class, no receiver-operating characteristic curves are presented (equivalently, the area under the receiver-operating characteristic curve metric was not calculated). The precision and recall of the imaging model were 0.77 and 0.89 on the amalgamated test set respectively. In comparison, we found that predictions made by consensus had a precision of 0.79 (range across raters: 0.70-0.85) and recall of 0.79 (range: 0.58-0.84). A Fleiss Kappa score of 0.74 was obtained for inter-rater agreement.

We acknowledge that the three senior neuroradiologists had as much time as required to make the decision. We also acknowledge that consensus readings cannot be obtained routinely in the clinic and therefore our clinical comparator is an optimal scenario which may not be reflected in a routine clinical setting. Nonetheless, based on the consensus predictions and range in inter-rater predictive performances, the presented model (which returns predictions immediately and only requires images alone) performs at least similarly to the consensus of three experts given the same imaging. We therefore expect that the proposed deep learning model could provide relevant information for routine clinical practice, by distinguishing those patients who are and are not expected to survive the eight-month window after radiotherapy completion. Studies validating imaging model performance in clinical settings are required to test this possibility.

**Table H1.** Comparison of imaging model performance to those obtained by expert clinical raters on the amalgamated test set (the amalgamation of the retrospective and prospective test sets). Performances are shown for predictions made by each reader based on the same images used as inputs to the reported imaging model. Performance of predictions based on consensus agreement across raters is also provided (based on mode/majority vote).

| **Description** | **Precision** | **Recall** | **F1** | **Specificity** | **NPV*^a^*** | **BAR*^b^*** | **Accuracy** |
| --- | --- | --- | --- | --- | --- | --- | --- |
| Imaging model | 0.77 | 0.89 | 0.83 | 0.83 | 0.92 | 0.86 | 0.85 |
| *Predictions from clinical experts, based on T1c and T2 post-radiotherapy imaging* | | | | | | | |
| Reader 1 | 0.85 | 0.58 | 0.69 | 0.93 | 0.77 | 0.75 | 0.79 |
| Reader 2 | 0.70 | 0.84 | 0.76 | 0.76 | 0.88 | 0.80 | 0.79 |
| Reader 3 | 0.70 | 0.74 | 0.72 | 0.79 | 0.82 | 0.76 | 0.77 |
| Consensus vote | 0.79 | 0.79 | 0.79 | 0.86 | 0.86 | 0.83 | 0.83 |

*^a^ NPV*: negative predictive value.

*^b^ BAR*: balanced accuracy rate.

*^c^ T1c*: contrast-enhanced T1-weighted MRI.

*^d^T2*: T2-weighted MRI.

**Appendix I. Further analysis of saliency maps**

**Appendix I1.** Analysis of saliency maps in relation to model predictions and tumor appearances

As a further analysis, we investigated the relationship between imaging model predictions, axially-selected slices of 3D saliency maps for the predicted outcome, and tumor-related regions in input MRI scans (including treated/resected tumour areas).

In Table I1 below, we show amalgamated test set patient numbers grouped by (a) prediction accuracy of survival outcome from the imaging model (accurately versus inaccurately classified) and (b) whether any visible heatmap points in axially-selected slices of volumetric saliency maps overlay tumor-related regions in T1c/T2 slices (intersection of (i) tumor regions in MRI scans, and (ii) visible “hot” heatmap area after thresholding to remove the lowest 10% of values).

|  | | **Intersection between salient heatmap points in automatically selected axial slices and tumor region(s) in T1c/T2 MRIs** | |
| --- | --- | --- | --- |
| **Predictive**  **accuracy**  **(imaging model)** |  | **Intersection** | **No Intersection** |
|  | **True positive**  **(short-term survival)** | 15 | 2 |
|  | **True negative**  **(long-term survival)** | 13 | 11 |
|  | **False positive** | 4 | 1 |
|  | **False negative** | 1 | 1 |

As noted in the main manuscript, saliency maps alone should not be interpreted as showing task-related features that are easily interpretable to human readers^13^. In this test set, 29/48 patients were long-term survivors (60.4%). It is conceivable, for example, that anatomical appearances reflecting long-term survival may be difficult to interpret. Indicators of longer survival may relate to the absence of features that signify more marked disease progression. Indeed, the tumor region in long term survivors (true negatives) appears less likely to contribute to the decision making (a relatively smaller portion of patients have MRI scans where the “hot” heatmap points intersects with the tumor).

**Appendix I2.** Saliency maps for patients misclassified as short-term survivors

We further reviewed all cases where patients with long-term survival after radiotherapy were mistakenly predicted as short-term survivors by the imaging model. There were five patients in the amalgamated test set in this category. All cases are presented in Figure I2 below to explore the possibility that these patients show signs of pseudoprogression, where the model mistakenly predicted short-term survival given treatment-related effects in MRIs. Since the maps do not consistently suggest that contrast-enhancing tumor regions contributed greatly to predictions, it is not evident that the mistaken predictions of short-term survival could relate to pseudoprogression in all cases. Further comments on the challenging and clinically important scenario of pseudoprogression are presented in the Discussion section of the main manuscript.


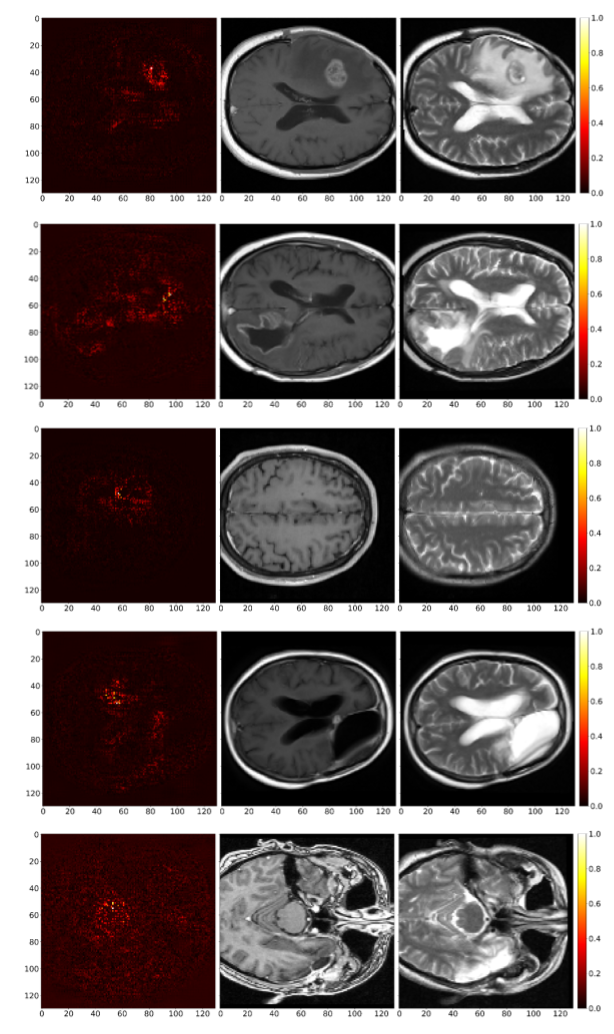


**Figure I2.** Visualization of saliency map, T1c, and T2 axial slices for all cases where patients with long-term survival were mistakenly predicted as short-term survivors.

*T1c*: contrast-enhanced. *T*_1_-weighted MR sequence. *T2*: *T*_2_–weighted MR sequence.

**References for Supplemental Material**

1. Weller M, Van Den Bent M, Tonn JC, Stupp R, Preusser M, Cohen-Jonathan-Moyal E, Henriksson R, Le Rhun E, Balana C, Chinot O, Bendszus M. European Association for Neuro-Oncology (EANO) guideline on the diagnosis and treatment of adult astrocytic and oligodendroglial gliomas. The Lancet Oncology 2017;18:e315–e329. doi: 10.1016/S1470-2045(17)30194-8

2. Davies J, Reyes-Rivera I, Pattipaka T, Skirboll S, Ugiliweneza B, Woo S, Boakye M, Abrey L, Garcia J, Burton E. Survival in elderly glioblastoma patients treated with bevacizumab-based regimens in the United States. Neuro-Oncology Practice. 2018;5(4):251-61. doi: 10.1093/nop/npy001

3. Bates A, Gonzalez-Viana E, Cruickshank G, Roques T. Primary and metastatic brain tumours in adults: summary of NICE guidance. BMJ. 2018;362. doi: 10.1136/bmj.k2924

4. Stupp R, Brada M, Van Den Bent MJ, Tonn JC, Pentheroudakis GE. High-grade glioma: ESMO Clinical Practice Guidelines for diagnosis, treatment and follow-up. Annals of oncology. 2014;25:iii93-101. doi: 10.1093/annonc/mdu050

5. Stupp R, Mason WP, Van Den Bent MJ, Weller M, Fisher B, Taphoorn MJ, Belanger K, Brandes AA, Marosi C, Bogdahn U, Curschmann J. Radiotherapy plus concomitant and adjuvant temozolomide for glioblastoma. New Engadebland Journal of Medicine. 2005;352:987-96. doi: 10.1056/NEJMoa043330

6. Stupp R, Hegi ME, Mason WP, Van Den Bent MJ, Taphoorn MJ, Janzer RC, Ludwin SK, Allgeier A, Fisher B, Belanger K, Hau P. Effects of radiotherapy with concomitant and adjuvant temozolomide versus radiotherapy alone on survival in glioblastoma in a randomised phase III study: 5-year analysis of the EORTC-NCIC trial. The Lancet Oncology. 2009;10:459-66. doi: 10.1016/S1470-2045(09)70025-7

7. Felsberg J, Rapp M, Loeser S, Fimmers R, Stummer W, Goeppert M, Steiger HJ, Friedensdorf B, Reifenberger G, Sabel MC. Prognostic Significance of Molecular Markers and Extent of Resection in Primary Glioblastoma Patients Molecular Markers in Glioblastoma Patients. Clinical Cancer Research. 2009;15(21):6683-93. doi: 10.1158/1078-0432.CCR-08-2801

8. Brown TJ, Brennan MC, Li M, Church EW, Brandmeir NJ, Rakszawski KL, Patel AS, Rizk EB, Suki D, Sawaya R, Glantz M. Association of the extent of resection with survival in glioblastoma: a systematic review and meta-analysis. JAMA oncology. 2016;2(11):1460-9. doi:10.1001/jamaoncol.2016.1373

9. Helseth R, Helseth E, Johannesen TB, Langberg CW, Lote K, Rønning P, Scheie D, Vik A, Meling TR. Overall survival, prognostic factors, and repeated surgery in a consecutive series of 516 patients with glioblastoma multiforme. Acta neurologica scandinavica. 2010;122(3):159-67. doi: 10.1111/j.1600-0404.2010.01350.x

10. Lamborn KR, Chang SM, Prados MD. Prognostic factors for survival of patients with glioblastoma: recursive partitioning analysis. Neuro-oncology. 2004;6(3):227-35. doi: 10.1215/S1152851703000620

11. Booth TC, Luis A, Brazil L, Thompson G, Daniel RA, Shuaib H, Ashkan K, Pandey A. Glioblastoma post-operative imaging in neuro-oncology: current UK practice (GIN CUP study). European radiology. 2021;31:2933-43. doi: 10.1007/s00330-020-07387-3

12. Paszke A, Gross S, Massa F, Lerer A, Bradbury J, Chanan G, Killeen T, Lin Z, Gimelshein N, Antiga L, Desmaison A. Pytorch: An imperative style, high-performance deep learning library. Advances in neural information processing systems. 2019;32:8026-8037

13. Adebayo J, Gilmer J, Muelly M, Goodfellow I, Hardt M, Kim B. Sanity checks for saliency maps. Advances in Neural Information Processing Systems. 2018;31
